# Eleven quick tips for finding research data

**DOI:** 10.1371/journal.pcbi.1006038

**Published:** 2018-04-12

**Authors:** Kathleen Gregory, Siri Jodha Khalsa, William K. Michener, Fotis E. Psomopoulos, Anita de Waard, Mingfang Wu

**Affiliations:** 1 Data Archiving and Networked Services, Royal Netherlands Academy of Arts and Sciences, The Hague, Netherlands; 2 National Snow and Ice Data Centre, Cooperative Institute for Research in Environmental Sciences, University of Colorado, Boulder, Colorado, United States of America; 3 College of University Libraries & Learning Sciences, The University of New Mexico, Albuquerque, New Mexico, United States of America; 4 Institute of Applied Biosciences, Centre for Research and Technology Hellas, Thessaloniki, Greece; 5 Research Data Management Solutions, Elsevier, Jericho, Vermont, United States of America; 6 Australia National Data Service, Melbourne, Australia; Genome Quebec, CANADA

This is a *PLOS Computational Biology* Education paper.

## Introduction

Over the past decades, science has experienced rapid growth in the volume of data available for research—from a relative paucity of data in many areas to what has been recently described as a data deluge [‎^1^]. Data volumes have increased exponentially across all fields of science and human endeavour, including data from sky, earth, and ocean observatories; social media such as Facebook and Twitter; wearable health-monitoring devices; gene sequences and protein structures; and climate simulations [‎^2^]. This brings opportunities to enable more research, especially cross-disciplinary research that could not be done before. However, it also introduces challenges in managing, describing, and making data findable, accessible, interoperable, and reusable by researchers [‎^3^].

When this vast amount and variety of data is made available, finding relevant data to meet a research need is increasingly a challenge. In the past, when data were relatively sparse, researchers discovered existing data by searching literature, attending conferences, and asking colleagues. In today’s data-rich environment, with accompanying advances in computational and networking technologies, researchers increasingly conduct web searches to find research data. The success of such searches varies greatly and depends to a large degree on the expertise of the person looking for data, the tools used, and, partially, on luck. This article offers the following 11 quick tips that researchers can follow to more effectively and precisely discover data that meet their specific needs.

- Tip 1: Think about the data you need and why you need them.
- Tip 2: Select the most appropriate resource.
- Tip 3: Construct your query strategically.
- Tip 4: Make the repository work for you.
- Tip 5: Refine your search.
- Tip 6: Assess data relevance and fitness -for -use.
- Tip 7: Save your search and data- source details.
- Tip 8: Look for data services, not just data.
- Tip 9: Monitor the latest data.
- Tip 10: Treat sensitive data responsibly.
- Tip 11: Give back (cite and share data).

## Tip 1: Think about the data you need and why you need them

Before embarking on a search for data, consider how you will use the desired data in the context of your overall research question. Are you seeking data for comparison or validation, as the basis for a new study, or for another reason? List the characteristics that the data must have in order to fulfil your identified purpose(s), including requirements such as data format, spatial or temporal coverage, availability, and author or research group. In many cases, your initial data requirements and the identified constraints will change as you progress with the search. Pausing to first analyse what you need and why you need it can lead to a more analytic search, save searching time and facilitating the actions described in Tips 2–6.

## Tip 2: Select the most appropriate resource

Directories of research-data repositories, such as re3data.org (http://www.re3data.org) and FAIRsharing (https://fairsharing.org), web search engines, and colleagues can be consulted to discover domain-specific portals in your discipline. Subject domain is but one criterion to consider when selecting an appropriate data repository. Various certification processes have also been implemented to help develop trustworthiness in repositories and to make their data-governing policies more transparent. For example, repositories earning the CoreTrustSeal (https://www.coretrustseal.org/about) Trustworthy Data Repository certification must meet 16 requirements measuring the accessibility, usability, reliability, and long-term stability of their data. Knowing what standards and criteria a repository applies to data and metadata provides more confidence in understanding and reusing the data from that repository.

Domain-specific portals provide ways to quickly narrow your search, offering interfaces and filters tailored to match the data and needs of specific disciplinary domains. Map interfaces for data collected from specific locations (see the National Water Information System, https://maps.waterdata.usgs.gov/mapper/index.html) and specific search fields and tools (see the National Centre for Biotechnology Information’s complement of databases, (https://www.ncbi.nlm.nih.gov/guide/all/) facilitate discovering disciplinary data. Other domain-focused repositories, such as the National Snow and Ice Data Centre (NSIDC, http://nsidc.org/data/search/), collect and apply knowledge about user requirements and incorporate domain semantics into their search engines to help data seekers quickly find appropriate data. Data aggregators, including DataONE (https://www.dataone.org) for environmental and earth observation data, VertNet (http://vertnet.org) and Global Biodiversity Information Facility (GBIF, https://www.gbif.org) for museum specimen and biodiversity data, or DataMed (https://datamed.org) for biomedical datasets, enable searching multiple data repositories or collections through a single search interface. Some portals may not provide data-search functionality but instead provide a catalogue of data resources. A notable example is the AgBioData (https://www.agbiodata.org/databases) portal, which lists links to 12 agricultural biological databases dedicated to specific species (e.g., cotton, grain, or hardwood), where you can directly search for data.

The accessibility of data resources is another important consideration. University librarians can provide advice about particular subscription-based resources available at your institution. Research papers in your field can also point to available data repositories. In domains such as astronomy and genomics, for example, citations of datasets within journal articles are commonplace. These references usually include dataset access information that can be used to locate datasets of interest or to point toward data repositories favoured within a discipline.

## Tip 3: Construct your query strategically

Describing your desired data effectively is key to communicating with the search system. Your description will determine if relevant data are retrieved and may inform the order of the hits in the results list. Help pages provide tips on how to construct basic and advanced searches within particular repositories (see for example Research Data Australia https://researchdata.ands.org.au—click on Advanced Search → Help). Note that not all repositories operate in the same manner. Some portals, such as DataONE (https://www.dataone.org), use semantic technologies to automatically expand the keywords entered in the search box to include synonyms. If a portal does not use automatic expansion, you may need to manually add various synonyms to your search query (e.g., in addition to ‘demography’ as a search term, one might also add ‘population density’, ‘population growth’, ‘census’, or ‘anthropology’).

If you are looking for data that span different disciplines, or if you cannot find a suitable domain repository and opt instead for a general search engine, learn how to make the most of search operators. The ‘site:’ operator, for example, restricts web domains for returned results. For example, using this search string

*sea level* *(site:.edu)*

will return results only from sites whose URLs end in.edu when using Google or Bing. You can find out what operators are supported by your selected search engine by searching for them directly. For example, the query ‘google search operators’ results in pages such as [‎^4^] describing all operators that can be used in your query. Constructing a query with search operators is like writing a program script: They allow you to precisely communicate what you are searching for, with the reward being the retrieval of more on-topic results.

## Tip 4: Make the repository work for you

Repository developers invest significant time and energy organizing data in ways to make them more discoverable; use their work to your advantage. Familiarize yourself with the controlled vocabularies, subject categories, and search fields used in particular repositories. Searching for and successfully locating data is dependent on the information about the data, termed metadata, that are contained in these fields; this is particularly true for numeric or nontextual data. Browsing subject categories can also help to gauge the appropriateness of a resource, home in on an area of interest, or find related data that have been classified in the same category.

Researchers can also register or create profiles with many data repositories. By registering, you may be able to indicate your general research data interests which can be utilized in subsequent searches or receive alerts about datasets that you have previously downloaded (see also Tip 7).

## Tip 5: Refine your search

In many cases, your initial search may not retrieve relevant data or all of the data that you need. Based on the retrieved results, you may need to broaden or narrow your approach. Apart from rephrasing your search query and using search operators, as discussed in Tip 3, facets or filters specific to individual repositories can be used to narrow the scope of your results. Refinements such as data format, types of analysis, and data availability allow users to quickly find usable data.

Examining results that look interesting (for example, by clicking on links for ‘more information’) can be a signal of the type of information that you find relevant. These results can then be linked to related ones (e.g., from the data provider, from different time series), and in subsequent searches, other results algorithmically determined to be related will be brought to the top of the results list.

## Tip 6: Assess data relevance and fitness for use

Conduct a preliminary assessment of the retrieved data prior to investing time in subsequent data download, integration, and analytic and visualization efforts. A quick perusal of the metadata (text and/or images) can often enable you to verify that the data satisfy the initial requirements and constraints set forth in Tip 1 (e.g., spatial, temporal, and thematic coverage and data-sharing restrictions). Ideally, the metadata will also contain documentation sufficient to comprehensively assess the relevance and fitness for use of the data, including information about how the data were collected and quality assured, how the data have been previously used, etc. Some data repositories such as the National Science Foundation’s Arctic Data Centre (https://arcticdata.io) enable the data seeker to generate and download a metadata quality report that assesses how well the metadata adhere to community best practices for discovery and reusability. Clearly, if none of your criteria for data are met, you may not wish to download and use the associated data.

Attention should also be paid to quality parameters or flags within the data files. Make use of a visualization tool or statistics analysis tool, if provided, to examine quality or fitness of data for intended use before downloading data, especially if the data volume is large and the dataset includes many files.

## Tip 7: Save your search and data-source details

Record the data source and data version if you access or download a data product. This may be accomplished by noting the persistent identifier, such as a digital object identifier (DOI) or another Global Unique Identifier (GUID) assigned to the data. Recording the URL from which you obtained the data can be a quick way of returning to it but should not be trusted in the long term for providing access to the data, as URLs can change. It is also a good practice to save a copy of any original data products that you downloaded [‎^5^]. You may, for example, need to go back to original data sources and check if there have been any changes or corrections to data. Registering with the data portal (as described in Tip 3) or registering as a user of a specific data product allows the repository to contact you when necessary. Such information may be needed when you publish a paper that builds on the data you accessed. If there are any errors found in the original data, registering with the data service allows them to contact you to see if there is an impact on any research conclusions that you have drawn from this data.

If you have registered with a portal, it may also be possible to save your searches, allowing you to resume your data search at a later time with all previously defined search criteria. Some portals use RESTful search interfaces, which means you can bookmark a results set or dataset and return to it later simply by going to the bookmark.

## Tip 8: Look for data services, not just data

The data you seek may be available only via an application programming interface (API) or as linked data [‎^6^]. That is, instead of a file residing on a server, the data that best suits your purposes is provided as a service through an API. Examples of such services include the climate change projection data available through the NSW Climate Data Portal (http://climatechange.environment.nsw.gov.au/Climate-projections-for-NSW/Download-datasets), in which data are dynamically generated from a simulation model; Google Earth Engine (https://earthengine.google.com); or Amazon Web Services (AWS) public datasets (https://aws.amazon.com/public-datasets/). Data made available from these services may not be searchable from general web search engines, but data services may be registered to data catalogues or federations such as Research Data Australia, DataONE, and other resources listed in re3data.org and FAIRsharing. Many repositories that host extremely large volumes of data such as sequencing, environmental observatory, and remotely sensed data provide access to tools, workflows, and computing resources that allow one to access, visualize, process, and download manageable subsets of the data. Often, the processing workflows that one might use to process and download a dataset can also be downloaded, saved, and used again in subsequent searches.

## Tip 9: Monitor the latest data

One of the most effective ways to identify new data submissions is to monitor the latest literature, as many journals such as *Nature*, *PLOS*, *Science*, and others require that the data underlying a publication also be published in a public (e.g., Dataverse https://dataverse.org, Dryad http://datadryad.org, or Zenodo https://zenodo.org) or discipline-based repository (e.g., EASY from Data Archiving and Networked Services [DANS] https://easy.dans.knaw.nl/, GenBank https://www.ncbi.nlm.nih.gov/genbank/, or PubChem https://pubchem.ncbi.nlm.nih.gov).

In addition, many domain-based repositories, such as environmental observatories and sequencing databases, are constantly accepting similar types of data submissions. Publishers and some digital repositories also offer alerting services when new publications or data products are submitted. Depending on the resource, it may be possible to set up a recurring search API or a Rich Site Summary (RSS) feed to automatically monitor specific resources. For example, the NSIDC offers a subscription service where new data meeting a list of user-generated specifications are automatically pushed to a location specified by the user.

## Tip 10: Treat sensitive data responsibly

In most cases, after you have located relevant data, you can download them straight away. However, there are cases, such as for medical and health data, endangered and threatened species, and sacred objects and archaeological finds, where you can only see a data description (the metadata) and are not able to download the data directly due to access restrictions imposed to protect the privacy of individuals represented in the data or to safeguard locations and species from harm or unwanted attention. Guidance with respect to sensitive data is available through the 2003 Fort Lauderdale Agreement (https://www.genome.gov/pages/research/wellcomereport0303.pdf), the 2009 Toronto Agreement (https://www.nature.com/articles/461168a) [7], the Australian National Data Service (http://www.ands.org.au/working-with-data/sensitive-data), and individual institutional and society research ethics committees.

Sensitive data are often discoverable and accessible if identity and location information are anonymized. In other cases, an established data-access agreement specifies the technical requirements as well as the ethical and scientific obligations that accessing and using the data entail. Technical requirements may include aspects such as auditing data access at the local system, defining read-only access rights, and/or ensuring constraints for nonprivileged network access. You can still contact the data owner to explain your intended use and to discuss the conditions and legal restrictions associated with using sensitive data. Such contact may even lead to collaborative research between you and the data owner. Should you be granted access to the data, it is important to use the data ethically and responsibly [8] to ensure that no harm is done to individuals, species, and culture heritages.

## Tip 11: Give back (cite and share data)

There are three ways to give back to the community once you have sought, discovered, and used an existing data product. First, it is essential that you give proper attribution to the data creators (in some cases, the data owners) if you use others’ data for research, education, decision making, or other purposes [9]. Proper attribution benefits both data creators/providers and data seekers/users. Data creators/providers receive credit for their work, and their practice of sharing data is thus further encouraged. Data seekers/users make their own work more transparent and, potentially, reproducible by uniquely identifying and citing data used in their research.

Many data creators and institutions adopt standard licenses from organizations, such as Creative Commons, that govern how their data products may be shared and used. Creative Commons recommends that a proper attribution should include title, author, source, and license [10].

Second, provide feedback to the data creators or the data repository about any issues associated with data accessibility, data quality, or metadata completeness and interpretability. Data creators and repositories benefit from knowing that their data products are understandable and usable by others, as well as knowing how the data were used. Future users of the data will also benefit from your feedback.

Third, virtually all data seekers and data users also generate data. The ultimate ‘give-back’ is to also share your data with the broader community.

## Conclusion

This paper highlights 11 quick tips that, if followed, should make it easier for a data seeker to discover data that meet a particular need. Regardless of whether you are acting as a data seeker or a data creator, remember that ‘data discovery and reuse are most easily accomplished when: (1) data are logically and clearly organized; (2) data quality is assured; (3) data are preserved and discoverable via an open data repository; (4) data are accompanied by comprehensive metadata; (5) algorithms and code used to create data products are readily available; (6) data products can be uniquely identified and associated with specific data originator(s); and (7) the data originator(s) or data repository have provided recommendations for citation of the data product(s)’ [11].
